# Biomaterial-Mediated Factor Delivery for Spinal Cord Injury Treatment

**DOI:** 10.3390/biomedicines10071673

**Published:** 2022-07-12

**Authors:** Filippo Pinelli, Fabio Pizzetti, Valeria Veneruso, Emilia Petillo, Michael Raghunath, Giuseppe Perale, Pietro Veglianese, Filippo Rossi

**Affiliations:** 1 Department of Chemistry, Materials and Chemical Engineering “Giulio Natta”, Politecnico di Milano, Via Mancinelli 7, 20131 Milan, Italy; filippo.pinelli@polimi.it (F.P.); fabio.pizzetti@polimi.it (F.P.); emilia.petillo@mail.polimi.it (E.P.); 2 Istituto di Ricerche Farmacologiche Mario Negri IRCCS, Via Mario Negri 2, 20156 Milan, Italy; valeria.veneruso@marionegri.it; 3 Center for Cell Biology and Tissue Engineering, Institute for Chemistry and Biotechnology (ICBT), Zurich University of Applied Sciences (ZHAW), 8820 Wädenswil, Switzerland; ragh@zhaw.ch; 4 Faculty of Biomedical Sciences, University of Southern Switzerland (USI), Via Buffi 13, 6900 Lugano, Switzerland; giuseppe.perale@usi.ch; 5 Ludwig Boltzmann Institute for Experimental and Clinical Traumatology, Donaueschingenstrasse 13, 1200 Vienna, Austria

**Keywords:** hydrogels, inflammation, nanoparticles, regeneration, spinal cord

## Abstract

Spinal cord injury (SCI) is an injurious process that begins with immediate physical damage to the spinal cord and associated tissues during an acute traumatic event. However, the tissue damage expands in both intensity and volume in the subsequent subacute phase. At this stage, numerous events exacerbate the pathological condition, and therein lies the main cause of post-traumatic neural degeneration, which then ends with the chronic phase. In recent years, therapeutic interventions addressing different neurodegenerative mechanisms have been proposed, but have met with limited success when translated into clinical settings. The underlying reasons for this are that the pathogenesis of SCI is a continued multifactorial disease, and the treatment of only one factor is not sufficient to curb neural degeneration and resulting paralysis. Recent advances have led to the development of biomaterials aiming to promote in situ combinatorial strategies using drugs/biomolecules to achieve a maximized multitarget approach. This review provides an overview of single and combinatorial regenerative-factor-based treatments as well as potential delivery options to treat SCIs.

## 1. Introduction

Spinal cord injury (SCI) is one of the most disabling events that involves the central nervous system [1,2,3], causing temporary or permanent loss of muscle function, sensation, or autonomic function in the parts of the body served by the spinal cord below the level of the injury. Depending on the location and the severity of the damage, the symptoms may vary, from numbness to paralysis, including bowel or bladder incontinence. Long-term outcomes also range widely, from full recovery to permanent tetraplegia or paraplegia. Globally, around 2.5 million people live with SCIs, and every year 180,000 novel cases are registered [4,5]. Obviously, SCI has a grave impact on the quality of life of the patients, and incurs heavy costs from both social and healthcare points of view [6,7,8]. From a pathophysiological perspective, the trauma starts with an acute phase (primary mechanical injury) as a consequence of compressive, stretching, or contusive trauma [9,10,11,12,13]. This phase is then followed by a multifactorial process (also known as secondary injury) that aggravates the tissue damage and, thus, worsens the condition of the patient [14], leading finally to variable motor dysfunction, chronic pain syndrome, and many other permanent and critical outcomes. Numerous in vivo studies in recent decades have furthered our understanding of the pathophysiological mechanisms of the secondary injury and their kinetics [15,16]. A core problem in SCI is impaired axonal regeneration, as reflected by the limited gene expression of many regeneration-associated proteins, such as Tα1-tubulin, c-jun, NCAM, GAP-43, CAP-43, ATF3, STAT3, or CREB [17]. In addition, there is a lack of key trophic factors in SCI lesions, such as brain-derived growth factor (BDNF), nerve growth factor (NGF), vascular endothelial growth factor (VEGF), neurotrophin 3 (NT-3), glial-cell-line-derived neurotrophic factor (GDNF), and insulin growth factor 1 (IGF-1) [18,19]

**Figure 1:**
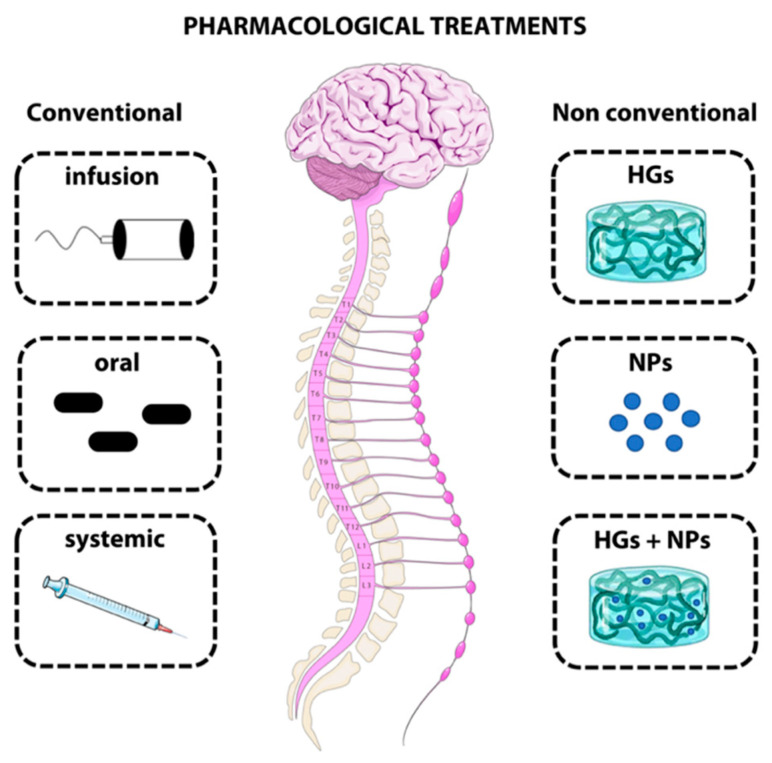
Schematic overview of conventional and non-conventional delivery strategies for SCI treatment.

Moreover, there is consensus that, in contrast to the embryonic phase and to what is known in axonal regeneration in amphibians, in mammalians the SCI lesion turns into an adverse environment for axon regeneration, thus fueling processes of the secondary injury. These processes include inflammation, glial scarring, and demyelination [15,20,21] (Figure 1). Inflammation includes processes of cellular response including the activation of microglia, with consequent morphological and molecular changes. In the injury site, a cascade of events starts, constituting secondary injury until the pathological changes come to a standstill, representing the chronic phase of SCI [22,23,24,25,26].

The main cellular actors are astrocytes, microglia, and oligodendrocytes. This scarring is able to produce signals (e.g., semaphorin 3 or tenascin) that can inhibit the regeneration of axons [27,28]. Neurite growth is also limited by specific proteins related to myelin, such as nogo, oligodendrocyte myelin glycoprotein, and myelin-associated glycoprotein [29,30]. This hostile environment is also exacerbated by the mechanism of inflammation, where microglia are activated, with consequent morphological and molecular changes, and move to the injury site, starting the cascade events typical of secondary injury until the complete stabilization (chronic phase) [23,31].

In recent years, two major strategies to curb the pathogenic cascade in subacute SCI have been proposed: neuroprotection, and neurodegeneration [32]. The first seeks to prevent neuronal degeneration by reducing the spread of tissue damage, while the second aims at rewiring neuronal connections and helping the regeneration of axons [33,34]. In this review, we focus our attention on different neuroprotective therapeutic strategies, such as administration of anti-inflammatory, antioxidant, or anti-apoptotic compounds, and on regenerative strategies that are dedicated to blocking myelin-associated neurite outgrowth inhibitors [35].

## 2. SCI Pathophysiology

Following a traumatic event that could take place after compression, laceration, or contusion of the spinal cord, the tissue leads to primary injury (acute neurological damage). However, most of the degeneration of the cord is due to the secondary injury that, in mammals, includes a wide spectrum of events, such as dysfunction of the blood–brain barrier, thrombosis, and neuronal death [14,36]. Among the different pathophysiological mechanisms, inflammation plays a key role, exacerbating the disease conditions.

The acute inflammatory response involves numerous cell types whose triggering signals are now partially discovered [37,38,39,40,41,42]. It is well known that an initial activation of resident microglia occurs, followed by recruitment of neutrophils, macrophages, dendritic cells, and B/T lymphocytes from the periphery [43]. Subsequently, astrocytes become reactive and show transient changes in phenotype, with regional reorganization, hypertrophy, and spread in the injured site [41,44]. Divergent roles are associated with different microglia/macrophage phenotypes: pro-inflammatory (M1 oversimplified) or anti-inflammatory (M2 oversimplified) cells—this classification is under current clarification because microglia/macrophages can show more than two polarization states [43,45,46]. Interestingly, M1-like response is rapidly induced and then maintained in the subacute and chronic phase after SCI, whereas M2 response is transient and limited to the subacute phase of the trauma [45,47]. On the other hand, astrocytes are not a uniform cell population, but present a complex spectrum of activation states with opposite phenotypes—some destructive (oversimplified A1), and others beneficial (oversimplified A2). They enjoy functionally relevant communication with immune cells (i.e., microglia [48,49]) involved in the spreading of the secondary injury. Recently, a close relationship has been demonstrated between microglia and astrocytes; it seems highly likely that microglia are capable of modulating astrocytes, and vice versa [48,49]. Microglia have been demonstrated to be activators of astrocytes by releasing some cytokines (interleukin 1 alpha (IL-1α), tumor necrosis factor alpha (TNF-α), and the complement component subunit 1q (C1q)), which are able to induce A1 astrocytes [48,49]. This suggests that the activation of microglia/macrophages and the associated inflammatory response could be a self-propelling mechanism of progressive inflammation by acting on the pro-inflammatory activated state of astrocytes and contributing to the SCI. Recently, Milich et al. exploited single-cell analysis to investigate cell heterogeneity at the injury site in a murine model of SCI [46]. This study revealed the presence of six different microglia subpopulations based on their transcriptional profiles: homeostatic microglia are present in uninjured mice, whereas at 1 DPI almost all microglial cells retrieved from the injury site shift towards an inflammatory and proliferative phenotype characterized by the upregulation of the *Msr1* and *Cdk1* genes [46]. Afterwards, between 3 and 7 DPI, a gradual conversion from the inflammatory signature towards [38,39,40,50,51] the homeostatic phenotype was observed [46]. The same paper demonstrated that, similarly to microglia, astrocytes also undergo a switch through different transcriptional profiles at different time points post-injury [46]. In addition, astrogliosis and microgliosis lead to proliferation, hypertrophy, and migration at the injured site, and many studies have characterized their contribution to scar formation [31,37,38,39,40,51,52,53,54]. Correlative investigations have shown that a glial scar was formed in the early hours and persisted for years after the injury [50]. A scar is a structured physical response to the lesion formed by cell migration and matrix deposition. Several populations, including fibroblasts derived from meninges and/or vessels, activated microglia/macrophages, activated astrocytes, pericytes, ependymal cells, and oligodendrocyte precursor cells, are able to migrate and proliferate, forming the scar [38,39,40,50,51]. A “concentric texture” of cells constituting the scar helps to narrow down when the injury took place—activated microglia/macrophages are placed closer to the lesion core, surrounded by oligodendrocyte precursor cells and pericytes [38,39,40,50,51], whereas hypertrophic astrocytes remain in the outer part of the scar (penumbra), reaching a higher density compared to naïve tissue [38,50].

However, the role of glial scarring is still highly debated; on the one hand it limits the disruption and amplification of the injury, while on the other it shows a detrimental effect, working against axonal regrowth, and acting as physical and chemical barrier [28]. Then, other phenomena, such as white matter demyelination, determine a deterioration of the pathological conditions until the achievement of a chronic condition [55].

The disease progression is also influenced by the presence of molecules with growth-inhibitory effects such as nogo-A, which causes growth inhibition and growth cone collapse by interacting with its receptor NgR1 [56,57]. Another inhibitory molecule is myelin-associated glycoprotein, produced by oligodendrocytes that limit axonal regrowth [58]. Spontaneous tissue regeneration is also limited by the presence of molecules belonging to the proteoglycan family, strongly involved in scar formation in SCI [59]. In the last year, Kwiecien et al. [3] confirmed prior studies on SCI demonstrating progressive astrogliosis that begins 1 day post-SCI through week 16. The numbers of macrophages peak at 1–4 weeks post-SCI, with their gradual decline by 12–16 weeks post-SCI, while astrogliosis progressively walls off the cavities of the injury, coinciding with a reduction in macrophage invasion. These findings suggest that persistent astrogliosis is associated with a gradual decrease in pro-inflammatory and increase in anti-inflammatory cytokines. All of these pathological mechanisms suggest that only a multitarget therapy able to simultaneously treat different mechanisms can have a good chance to reach clinical practice.

## 3. Drug Delivery to the Spinal Cord: The Role of Biomaterials in SCI Treatment

The pathogenic cascade of subacute SCI is largely localized in the spinal cord, and it follows that all of the abovementioned major therapeutic strategies to curb the pathogenic cascade in subacute SCI rely on localized, precise delivery of drugs and factors. Herein lies the specific challenge: The bony protective armor of the spinal cord aside, the conventional delivery of drugs to the damaged cord is highly limited by the presence of the blood–spinal cord barrier (BSCB)—a semipermeable interface of specialized small blood vessels that surround the spinal cord. It is indeed known that most therapeutic agents cannot cross this barrier if they are administered orally, systemically, or into the epidural space [60]. As known from pain therapy in cancer patients, alternative strategies can involve intrathecal administration, using catheters or minipumps, with several associated disadvantages, such as surgery-related side effects and the need to refill the pump [61].

In recent decades, novel targeting approaches have been proposed to overcome these limitations, such as the use of biodegradable carriers [62,63,64]. Most recently, in SCI treatment, several preclinical studies were carried out on two extremely promising biomaterial categories: hydrogels (HGs) and nanoparticles (NPs) [65,66,67].

These medical devices can carry a large variety of therapeutic agents (e.g., drugs, neurotrophins, and antibodies) and release them locally at the injury site [68]. An overview of the conventional and non-conventional treatments is presented in Table 1 and Figure 1.

### 3.1. Hydrogels

Hydrogels are 3D crosslinked networks of hydrophilic polymers able to retain a large amount of water without dissolving [69]. Crosslinking can be of two types: physical or chemical. Physical crosslinking corresponds to physical interactions, such as simple entanglement, while chemical crosslinking is related to covalent interactions. Polymers can have synthetic or natural origins. On the one hand, synthetic polymers can guarantee high tunability in terms of composition, degradation, and functionalization. On the other hand, natural polymers are able to provide structures that can stimulate cell response, and are generally less inflammatory and toxic [70]. Due to their elastic nature, HGs can be injected at the injury site, filling the SCI cavity, where they can release active agents and cells [71,72,73]. Furthermore, a promising property of HGs is the possibility of direct in situ gelation. Its advantages are related to reducing several drawbacks of classical surgery that can exacerbate the patient’s condition [74]. Moreover, their swelling ability, degradation rates, and mechanical properties make hydrogels ideal tools not only for the delivery of factors and small molecules, but also for hosting cells which, in turn, serve as drug delivery units [1,75,76].

In recent years, great attention has been dedicated to HGs in SCI—especially for drug or cell delivery [71,77]. HGs can indeed be loaded with drugs and sustain their release over time [78,79]. The release of small molecules has the problem that it can be uncontrolled (burst release); thus, different strategies should be considered with respect to the physical loading within the 3D network [74,80]. For example, curcumin can ameliorate SCI once it is released constantly over time from a dynamic reversible hybrid hydrogel made of fluorenylmethoxycarbonyl protecting group (Fmoc)-grafted chitosan and Fmoc peptide [81]. In this case, the interactions between the matrix and the drug molecules can sustain the release over time. With regard to bio-compounds’ release, several in vivo studies have shown that HGs can be designed for a sustained release of neurotrophins into the SCI lesion [82,83,84,85].

Recent studies have indeed demonstrated that the administration of (exogenous) neurotrophins such as NT-3, NT-4/4, NGF, BNDF, and glial-cell-line-derived neurotrophic factor (GDNF) promotes regeneration in SCI [86]. In recent years, different methods have been used to administer them, including systemic administration, direct injection, or intrathecal infusion pump. However, as already pointed out, all of these methods show many disadvantages, such as the inaccessibility of the BSCB, no control of the release, and problems due to surgery (such as placement of a catheter, creation of a pouch for a pump, etc.). In order to solve these problems, HGs were chosen as promising biomaterials that can sustain the release of growth factors directly at the injury site—a winning point when also considering their short bioavailability [87,88,89]. Indeed, HGs demonstrated good ability to preserve the bioactivity of GDNF [90], NT-3 [91,92], BDNF [93,94], and fibroblast growth factor-2 (FGF-2) [95,96].

Growth factors can be also immobilized in different gels (e.g., in silk protein nanofiber hydrogels [97]) with hierarchical anisotropic microstructures to provide multiple physical and biological cues. The maintained bioactivity of the growth factors inside the hydrogels can regulate the neuronal/astroglial differentiation of neural stem cells. The aligned microstructures can facilitate cell migration and orientation, which then stimulate neuroregeneration. The release of growth factors can also be prolonged over time using soft thermosensitive electroactive HGs combined with functional electrical stimulation [98]. An alternative method able to guarantee a great amount of trophic factors at the injury site is represented by the use of transplanted cells loaded within HGs—the so called “medicinal cells approach” [99,100,101]. Indeed, one hypothesis validated by many studies [102,103,104] is that stem cells can regulate the delivery of trophic factors. Moreover, a key advantage of loading cells within HGs is that they are confined, overcoming the problems of uncontrolled differentiation after transplantation and adverse immune response [104].

**Figure 2:**
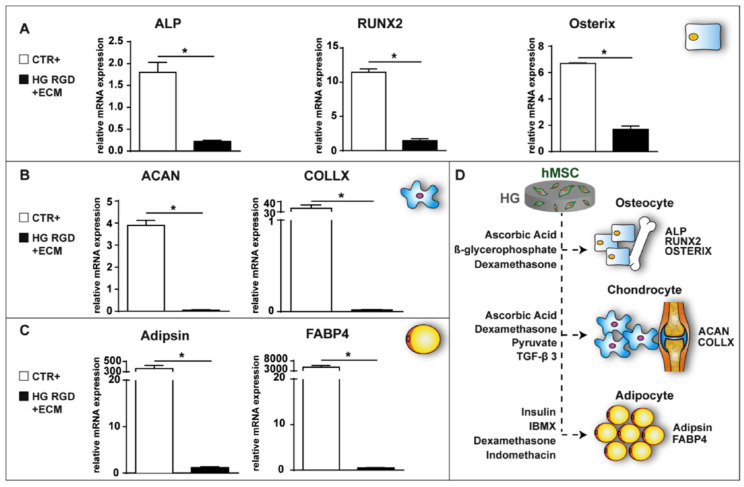
mRNA analysis of mesenchymal stem cells encapsulated within an agarose–Carbomer HG. (**A**–**C**) Graphs representing the expression of specific genes related to three differentiation lineages: alkaline phosphatase (ALP), runt-related transcription factor 2 (RUNX2), and osterix for osteogenic differentiation; aggrecan (ACAN) and collagen type X (COLLX) for chondrogenic differentiation; and adipsin and fatty-acid-binding protein 4 (FABP4) for adipogenic differentiation. Stem cells encapsulated within the HG for 21 days are compared to the positive control, represented by stem cells loaded in the HG and treated with specific differentiating media for 21 days. Data are expressed as the fold change compared to steady-state undifferentiated stem cells (negative control). (**D**) A representative cartoon of the three lineage commitments of stem cells (osteocytes, chondrocytes, and adipocytes), with respective principal pro-differentiating stimuli: ascorbic acid, b-glycerophosphate, and dexamethasone to induce osteogenic differentiation; ascorbic acid, dexamethasone, pyruvate, and TGF-b 3 to induce chondrogenic differentiation; insulin, 3-isobutyl-1-methylxanthine (IBMX), dexamethasone, and indomethacin to induce adipogenic differentiation. * *p* < 0.05, *n* = 3. Reprinted with permission from [109].

Different kinds of HGs were used for this purpose, like a system based on thiol-functionalized hyaluronic acid and thiol-functionalized gelatin that can create a neuroregenerative environment for transplanted oligodendrocyte progenitor stem cells [105]. Similarly, HGs from 2-hydroxyethyl methacrylate or 2-hydroxypropyl methacrylamide can reduce the lesion after being loaded with bone marrow stem cells [106]. In a chronic SCI model, HGs of Arg-Gly-Asp-*N*-(2-hydroxypropyl)-methacrylamide with mesenchymal stem cells were able to improve the infiltration of myelinated axons and astrocytes, reducing scarring and ameliorating the behavioral outcome [107,108]. HGs made of synthetic (Carbomer 974p) and natural components (agarose) can present the advantages of both of these polymer categories, and enable proper viability and release of active factors from stem cells in vitro and in vivo [109,110]. Moreover, they can maintain their stemness, avoiding differentiation in undesired cell populations such as osteocytes, adipocytes, or chondrocytes, as shown in Figure 2.

Some studies have dedicated a lot of attention to the specific types of molecules delivered from stem cells, such as human chemokine (C–C motif) ligand 2 chemokine (CCL2) secreted from human mesenchymal stem cells [104], the release of which from HGs can regulate macrophage recruitment and convert them to the neuroprotective phenotype M2, showing good improvements in motor performance in rodent SCI models [62,104]. Even if the strategy to load only factors and not cells is very interesting, the multitude of molecules released from cells cannot be easily simulated.

**Figure 3:**
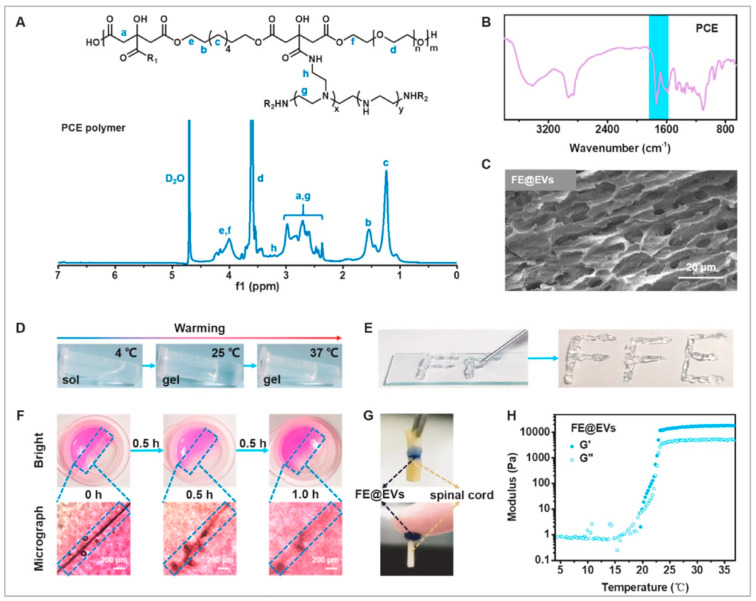
Physicochemical structure and multifunctional properties of the FE@EVs hydrogel. (**A**) ^1^H NMR spectra of the PCE polymer; (**B**) FTIR spectra of the FE hydrogel; (**C**) SEM image of the FE@EVs hydrogel. (**D**) The sol–gel transition of the FE@EVs hydrogel with temperature changes. (**E**) The photographs of the FE@EVs hydrogel through the needle. (**F**) The photographs of the FE@EVs hydrogel placed for a while after being cut off (hydrogel in blue dottex box). (**G**) The photographs of the FE@EVs hydrogel adhering to spinal cord (black arrow: FE@EVs, green arrow: spinal cord). (**H**) The G′ and G″ changes of the FE@EVs hydrogel at 4–38 °C. Reprinted with permission from [114].

Indeed, another key aspect that has recently showed promising results is represented by extracellular vesicles—microvesicles and exosomes delivered from cells [111]. They can be considered as mediators in cell communication that can mimic the action of stem cells carrying active molecules to the damaged cells [112,113]. The use of stem cells to provide extracellular vesicles is a good strategy, but their uncontrolled release and problems in their preservation are big issues [114]. A possible solution could be represented by the fabrication of an injectable adhesive anti-inflammatory F127-polycitrate-polyethyleneimine hydrogel (FE) with sustainable and long-term extracellular vesicle delivery (FE@EVs) that can improve motor functional recovery after SCI (multifunctional properties represented in Figure 3). This delivery can suppress scar formation, reduce inflammation, and promote neuroregeneration and remyelination.

### 3.2. Nanoparticles

In recent decades, advances in nanomedicine have provided several breakthroughs, and ensured the widening of applications in drug development and delivery—especially with the employment of nanoparticles (NPs) [115,116,117]. Polymeric nanoparticles have been shown to provide advantages in drug delivery by enhancing release kinetics, their targeting and, therefore, their concentration at the desired site, reducing systemic side effects [118,119]. Numerous polymeric nanoparticle species—variable in terms of size, hydrophilicity, and functionalization—have been developed to meet specific therapeutic needs [120,121,122], and following the state-of-the-art safety-by-design paradigm to ensure the highest biocompatibility and lowest toxicity [123]. Moreover, the great interest that is given to nanoparticles today must be sought in the capacity of these systems to pass biological barriers, entering and diffusing inside cells [124]. In this context, this kind of approach employing polymeric nanosystems—such as polymeric NPs, micelles, and nanowires—as vehicles for targeted therapies has proven to be very effective in the treatment of SCI [125,126]. Chemically conjugated, functionalized, and loaded nanoparticles are certainly one of the clearest examples of the employment of these devices in the treatment of this pathology and its inflammatory state [14]. For example, nanoparticles made of ferulic acid and glycol chitosan have been demonstrated to be able to reach the lesioned spinal cord and cause neuroprotection and functional restoration during systemic administration tests in an SCI rat model [127].

Similarly, the intravenous injection of nanoparticles containing prostaglandin E(1) has been shown to cause a reduction in the lesion cavity volume, promoting the recovery of motor dysfunction [128]. Another strategy reported in the literature for promoting the recovery of locomotor function and reducing the levels of the inflammatory state of the tissues is based on the conjugation of the cell-penetrating HIV trans-activator of transcription peptide of human serum albumin nanoparticles to obtain a delivery system for tetramethylpyrazione—an anti-inflammatory and antioxidant drug that could be internalized by neutrophils and delivered to SCI lesions sites [129]. This approach guarantees reduction in the inflammation state, and even the release of oxidative-stress-related factors that play an important role in the pain state of the pathology. Another approach—verified through assays in a clinically relevant rat SCI model—to reduce the induced oxidative damage during the secondary injury process of SCI is represented by the use of lipid–polymer nanoparticles with reactive oxygen species (ROS)-scavenging ability to eliminate these species for the lesion sites and, thus, reduce the long-term secondary injury [130].

The nanoparticles used for drug delivery applications are also frequently used to optimize the delivery of anti-inflammatory drugs. For example, this has been demonstrated with methylprednisolone (MP) loaded in PLGA NPs administered in situ, which ensured higher pharmacological efficacy compared to conventional routes of administration, and reduced tissue damage and the subsequent inflammatory state, improving the treatment results in an SCI rat model [126]. Similarly, micellar structures made of poly(ethylene oxide)-poly(propylene oxide)-poly(ethylene oxide) have been demonstrated to be effective in increasing the bioavailability of MP in the injured spinal cord. As already mentioned, systemic administration routes generally present limitations due to the presence of various barriers, such as the blood–spinal cord barrier (BSCB), which strongly limits the molecules and systems that can access the central nervous system [131,132,133]. Recently, carbohydrate-polymer-based nanoparticles, formed by polymerization of small-length sugars, have been deeply considered for intranasal drug delivery as an alternative to systemic administration [134]. These NPs are mucoadhesive and, therefore, no limitations of this route of administration are faced. Moreover, carbohydrate polymers and the surface functionalization of the systems with endogenous substances such as folic acid can improve the site-specific drug delivery to the brain, making them ideal candidates for improving the brain targeting and the drug pharmacokinetics. On the other hand, probably the most common means of administration of these devices is the direct injection of the NPs suspension in the injured site of the spinal cord, which enables all of the biological barriers of the body to be overcome and allows high targeting efficiency. For example, poly-lactic-co-glycolic acid (PLGA) NPs loaded with glial-cell-derived neurotrophic factors, directly injected into the damaged spinal cord to target neural and glial cells, have been reported to increase the neuronal survival and improve the motor locomotion [135].

Unfortunately, the NPs injected directly into the regional sites without any support often leave the zone of injection, and this strongly reduces the efficacy of the treatment, involving other sites that should not be affected by the drugs. Because of these issues, many efforts have reported to associate a properly designed device with NPs able to provide targeted therapy, to maximize the efficacy of the treatment and the targeting of the drugs without involving different bodily tissues. A clear example of this strategy was reported by Kang et al., who demonstrated how PLGA NPs loaded with fibroblast growth factor-2 (FGF-2) and embedded in a biopolymer blend of hyaluronan and methylcellulose implanted into the damaged spinal cord were able to enhance the endogenous angiogenic response of the body [136]. Similarly, the in situ delivery of this drug has been demonstrated to be optimized by the encapsulation of methylprednisolone in the PLGA NPs, and the whole system was subsequently entrapped in an agarose hydrogel and implanted at the site of the lesion, with an efficient release of active molecules and reduction in the early inflammation stage of the pathology.

It must be highlighted how, especially in the recent years, nanomaterials have been recognized as valuable devices for SCI treatment, and their neuroprotective efficacy has been widely investigated [137]. For example, the intravenous injection of micelles composed of self-assembled monoethoxy poly(ethylene glycol)-poly(D,L-lactic acid) deblock copolymer has been demonstrated to efficiently recover the locomotor function and reduce the lesion volume and inflammatory state in an SCI rat model [138]. Similarly, Zhou et al. designed click-chemistry-conjugated protein–drug micelles through the conjugation of ferrostatin-1 and didibenzocyclooctyne moieties to amphiphilic polymers, followed by click chemistry assembly with pH-responsive azido-linker-modified acidic fibroblast growth factor (aFGF) [139]. It is well known that acidic fibroblast growth factor participates in complex anti-inflammatory processes that confer neuroprotection and result in reduced scar formation during SCI [140]. Zhou et al. discovered that its release together with ferrostatin-1 through micelles can cause significant improvements in neural and motor recovery in the acidic SCI microenvironment, resulting in anti-ferroptotic and anti-inflammatory activities [139].

**Figure 4:**
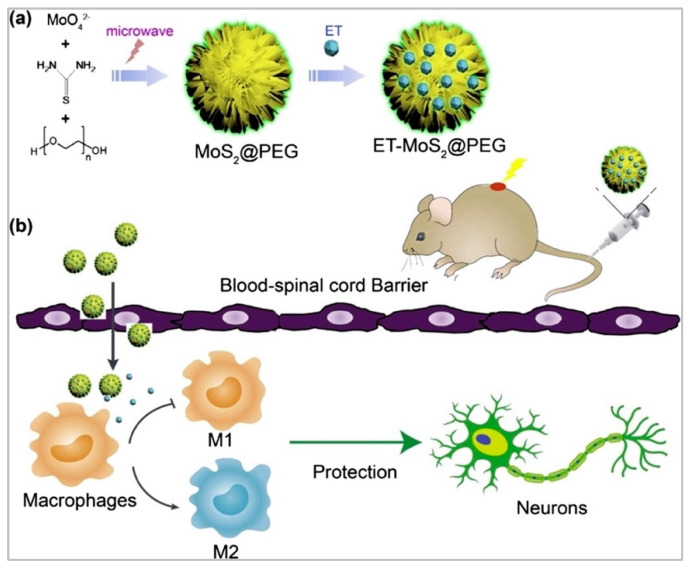
Schematic illustration of (**a**) the synthesis procedure for ET-MoS2@PEG nanoflowers, and (**b**) their application in SCI treatment as anti-inflammation devices. Reprinted with permission from [144].

Micelles can be employed even to promote the self-assembly of polymeric systems and subsequent sustained drug release, as reported by Wang et al. for E-selectin-targeting sialic acid–polyethylene glycol–poly (lactic-co-glycolic acid) assembly for delivering hydrophobic minocycline to achieve combinational therapy for SCI [141,142]. Great results in terms of inhibition of inflammatory response and neuronal protection have been obtained working with nanovesicles derived from macrophage membranes, which encapsulate sodium alginate and naloxone, and reduce the free Ca^2+^ concentration at the SCI site, which faces overloading after the primary injury and, hence, causes inflammation and neuronal apoptosis [143]. Anti-inflammatory treatment in spinal cord injury has been also reported with molybdenum disulfide poly (ethylene glycol) (PEG) nanoflowers for the loading of etanercept (ET) [144]. The drug loading and release ability of these devices has been characterized in vitro, and its ability in the inhibition of the expression of M1-related pro-inflammatory markers has been demonstrated together with the promotion of M2-related anti-inflammatory marker levels. The schematic illustration of the preparation and application of these nanoflowers is reported in Figure 4.

Nanowired materials are another valuable example of devices able to guarantee neuroprotective effects as delivery systems in SCI treatment. Tian et al. reported the design of TiO_2_ nanowires to increase the bioavailability of neuroprotective and anti-inflammatory drugs and improve their efficacy by achieving a higher concentration of the drug in the injured tissue [145]. As mentioned before, the interest in NPs as delivery tools in cell-targeted therapy is strictly related to their ability for entering specific cells, exploiting permissive pathways or receptors. Once internalized, NPs can release drugs in situ, with improved therapeutic efficacy, and avoiding conventional issues such as degradation or efflux of the active molecules. This strategy has been successfully applied in SCI treatment for the internalization of NPs by a specific endocytic/phagocytic activity of the macrophagic cells after different stimuli, exploiting them as Trojan horses [146]. Indeed, it is widely reported that microglia and macrophages, after traumatic events, assume phagocytic activity and, because of this, NPs are a valuable tool for drug targeting. Examples of this strategy have been reported in the literature by Cerqueira et al. and Papa et al. Cerqueira et al. designed surface-engineered carboxymethyl chitosan/polyamidoamine dendrimer NPs able to deliver MP into microglial cells, promoting controlled and selective release of the drug at the injured site [147]. On the other hand, in the work of Papa et al., NPs loaded with an anti-inflammatory drug (minocycline) were employed for the selective treatment of inflammatory cells. Specifically, non-biodegradable poly(methylmethacrylate) [148] and biodegradable poly-𝜖-caprolactone (PCL) [149] NPs were selectively internalized by microglia/macrophages, and minocycline-loaded PCL-based NPs were able to modulate the activation of microglia/macrophages in vitro and in vivo, reducing their proliferation. This selective delivery into cells was demonstrated to be more efficient compared with free delivery of the same molecule. Similarly, the same author proposed a functionalized PEG–PEI nanogel for selective treatment of activated astrocytes in spinal cord injury, limiting the phagocyte problem of macrophage/microglia [150].

**Figure 5:**
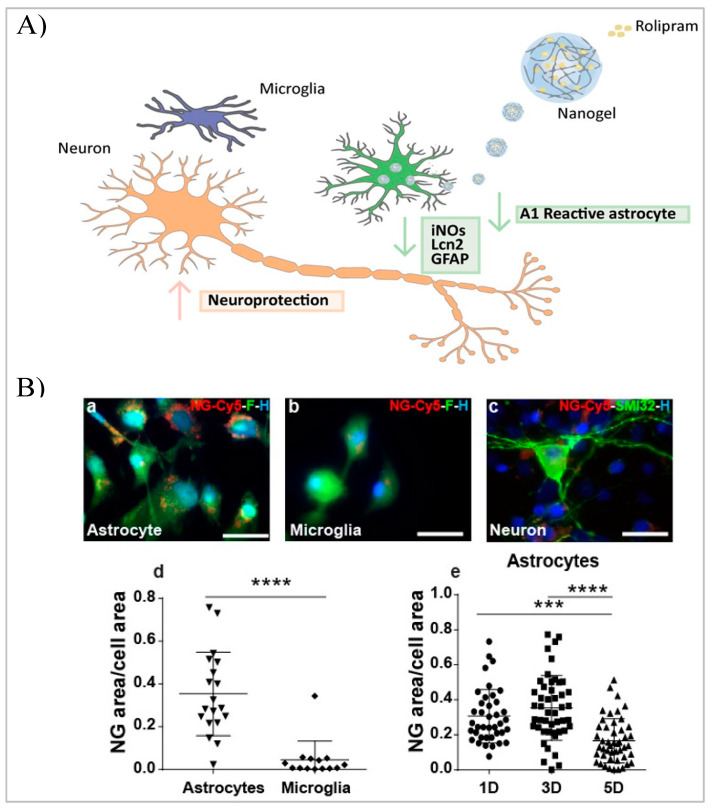
(**A**) Schematic representation of loaded nanogels’ internalization inside astrocyte cells, and subsequent neuroprotective effect. (**B**) Characterization of nanogels’ uptake in primary cultures of (**a**) astrocytes, (**b**) microglia, and (**c**) neurons. (**d**) The quantification of the nanogel uptake in activated astrocytes and microglia shows a higher degree of internalization in the former. (**e**) The quantification 1, 3, and 5 days after exposure shows a reduced signal due to the degradation of the nanovectors. Scale bar 25 μm. Statistical significance: *** *p* ≤ 0.001; **** *p* ≤ 0.0001. Reprinted with the permission of the American Chemical Society [151].

In vitro experiments showed how the internalization in the cells was mediated by a clathrin-dependent endocytic process, after which they underwent lysosomal degradation and subsequent release of active molecules with potential therapeutic efficacy. Vismara et al. applied this kind of device for the selective delivery of rolipram—an anti-inflammatory drug—in activated murine or human astrocytes [151]. These systems were able to limit the inflammatory response in A1 astrocytes, reversing the toxic effects of pro-inflammatory astrocytes on motor neurons in vitro, with advantages compared to conventional anti-inflammatory therapies. In Figure 5, the schematization of this nanogel therapy is reported, together with the characterization and quantification of its selective internalization inside the cells of the central nervous system.

However, in addition to those important results obtained from in vitro and in vivo studies, a critical issue frequently faced when using NPs is the safety of the nanostructured devices proposed as delivery systems. Their biocompatibility and efficacy are influenced by various features and parameters—such as size, shape, chemistry, solubility, and surface area [152,153]—and because of this, deeper investigations of these factors are mandatory before translation to clinical trials and medical practice [123,154].

### 3.3. Combinatorial Therapies

Even if theoretical studies on secondary injury are well supported by experimental evidence, the results of clinical trials on SCI still present disappointing results [155]. One of the reasons could be that the treatments proposed are directed only to specific mechanisms, not considering that SCI is a dynamic disease where the different physiopathological mechanisms do not take place simultaneously, and so it is reasonable to think that different targets should be addressed simultaneously at different times. Following this direction, several studies that use combinatorial treatments can be found in preclinical models. Indeed, recent studies are dedicated to multitherapeutic compounds that can efficiently target different mechanisms of the secondary injury [60]. Many of them propose the use of biomaterials that can release combinatorial therapies at the target site.

To better rationalize the possibilities, the combinatorial SCI therapies can be divided in four categories: (i) different growth factors directed to neuronal survival, axonal regrowth, and promotion of plasticity [156]; (ii) different drugs [157]; (iii) transplanted stem cells with different neurotrophic factors; or (iv) cells with trophic factors and biomaterial scaffolds [158]. In this context, biomaterials can work as substrates for cell transplantation, drive axonal regrowth, fill the cavity at the injury site, and act as drug reservoirs that can be released with controlled and sustained kinetics. Biomaterials can also be used to simultaneously counteract scar formation by releasing chondroitinase ABC and help in tissue regeneration [159]. Moreover, interesting outcomes arise from the combination between rehabilitation and pharmacological administration. Different studies have demonstrated that this combination can help to re-establish gait in transected murine models [160]. Moreover, the combination between chondroitinase ABC and rehabilitation can promote functional recovery in SCI [161]. Musieko et al. described the possibility of combining pharmacological treatment with epidural stimulation and rehabilitation to restore locomotion in murine models [162]. Even though recent studies have demonstrated that this combination is beneficial, the mechanisms behind the associated locomotor improvements are still debated [163]. Some more considerations regard the fact that in many studies the active compounds (e.g., growth factors, trophic factors, or drugs) are administered systemically, with consequent limited biodistribution. The barrier can indeed strictly permit the entrance of molecules to the spinal cord only to extremely small drug molecules [164]. Even if the barrier is partially destroyed in SCI, it is difficult to determine the amount of drug that can enter the cord, meaning that high doses may be needed to ensure an effective protection. Another key point is that with systemic administration the treatments cannot be selective and, thus, for example, the use of different trophic/growth factors may affect different cells simultaneously, and this may result in some adverse reactions, such as the alteration of responsiveness in the spinal circuitry [165,166,167]. Moreover, if used as single doses, neurotrophic factors cannot maintain constant biological efficacy, with consequent limited outcomes that should require multiple administrations [168]. All of these findings suggest that even if the combinatorial therapies are promising, they need to be improved, and biomaterials can play a pivotal role. Indeed, Hwang et al. used a scaffold made of PCL loaded with stem cells and NT-3 to bridge the cavity in a hemisected SCI model [169]. A similar strategy is represented by the combination of human fetal neural stem cells loaded within a polymeric scaffold together with serotonin [170]. This could lead to reductions in tissue atrophy and astrocytic activity, increasing axons’ ingrowth after scaffold implantation. Another combinatorial approach [171] regards the use of adult-brain-derived neural stem/progenitor cells together with recombinant rat-platelet-derived growth factor-A.

In order to ensure proper release kinetics, the growth factor is not simply loaded, but is covalently linked to a hyaluronan-based hydrogel. In addition, agarose hydrogels embedded with lipid microtubes were also used to sustain the simultaneous release of both Rho GTPases and BDNF [94]. This study demonstrates that the simultaneous alteration of multiple axonal responses can represent a promising approach to sustain spinal cord regeneration.

**Figure 6:**
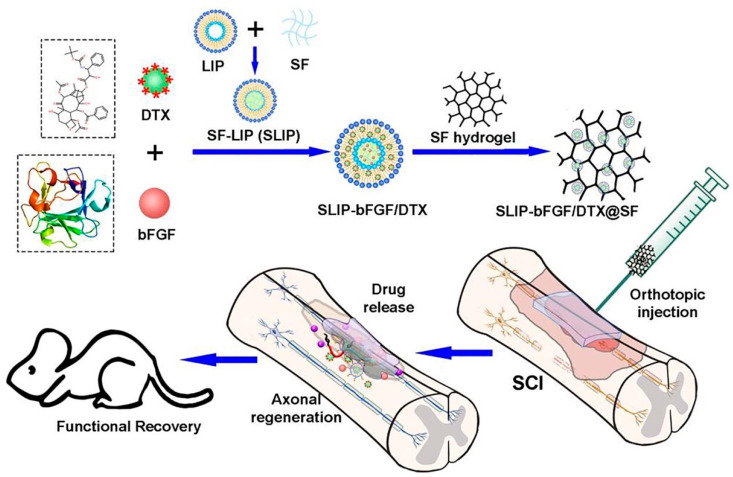
Schematic diagram of the injectable liposome–silk fibroin composite hydrogel as an in situ multiple drug delivery system for the treatment of SCI. Reprinted with permission from [173].

HGs can also be used to simultaneously release drugs and different growth factors with or without cells embedded in the 3D network [172]. Following this strategy, the synergistic release of methylprednisolone sodium succinate and growth factors can protect axons and tissues from secondary injury, and promotes scar-boundary- and cavity-free wound healing, resulting in permissive bridges for axonal regrowth [2]. Recent investigations have reported that docetaxel (DTX) can improve axonal regeneration, while FGF can regulate plasticity and neuronal survival after SCI. These can be loaded in a liposome (LIP) with a silk fibroin (SF) hydrogel core (SLIP) for their simultaneous release (Figure 6). This combination therapy was shown to have the ability to ameliorate various key pathological mechanisms [173]. Indeed, docetaxel is able to promote microtubule stabilization and stimulate axonal growth, while FGF can reduce the cavity area and neuronal loss, creating a good environment for neuroregeneration.

To guarantee proper release of hydrophobic and hydrophilic drugs, a good strategy is represented by the use of NPs together with HGs. In particular, hydrophobic drugs can be loaded within NPs and then immersed in a polymeric drug solution that can then create a composite HG after gelation. In this framework, the use of paclitaxel and minocycline was shown to reduce inflammation and decrease scar tissue [174]. Moreover, different drugs can be loaded in the same polymeric system, e.g., cetuximab and FTY720 together with stem cells [175]. The injectable system can improve the proliferation and neuronal differentiation of stem cells, and limit the astrocytic differentiation of stem cells.

### 3.4. Perspectives and Future Challenges

Despite very promising results, some issues should be solved before these methods reach clinics [176,177]. First of all, toxicity: detailed studies are necessary to confirm the degradation of hydrogels into non-toxic byproducts. Moreover, other challenges should be overcome during the translation process, such as hydrogels’ fabrication and storage, cost, and regulatory complexity. Indeed, their high water content makes sterilization extremely difficult, and sterility should be ensured for all manufacturing processes and raw materials. If stored in a dry state, to prevent premature degradation, the treatment used must guarantee that both its structure and drug bioactivity are unaltered. On the other hand, if maintained in a wet state, the storage and transport conditions should minimize water evaporation and unwanted drug loss. Moreover, regulatory concerns can also be a big obstacle. Indeed, a drug-releasing hydrogel is considered to be a combination product, and its regularity approval often takes longer with respect to the neat hydrogel. Thus, the high costs together with limited patent protection can be an issue for their commercial viability.

## 4. Conclusions

SCI’s physiopathology is an extremely disabling disease that heavily affects the life of the patients. As previously described, it is the result of a primary injury that is then followed by a secondary one, commonly known as the main cause of post-traumatic neural degeneration. Secondary injury involves different mechanisms, all of which play a role in the progressive loss of locomotor performance and tissue degeneration. Unluckily, different therapeutic treatments have produced only modest results when translated to clinical trials. A possible reason for this could be represented by the limitations of systemic drug administration due to BSCB restrictions and uncontrolled release rates of the active agents. To overcome these critical issues, researchers are looking toward the use of biomaterial-based delivery tools (e.g., HGs and NPs) to optimize SCI treatments. Various strategies have been proposed, as investigated in this review article, able to carry a large variety of therapeutic agents and release them locally. The main advantage in using this kind of devices is related to the localization of the therapies at the target site. As discussed, the use of nanosystems can ensure targeted release directed to specific cell lines, taking advantage of the selectivity of properly formulated devices. Similarly, HGs can be injected at the injury site, filling the SCI cavity and releasing in active agents and cells situ. Moreover, gels, thanks to their properties, are ideal tools not only for the delivery of factors or active molecules, but also for hosting cells and serving as drug delivery units, and because of this they are often combined with nanoparticles to increase their efficacy and confine their action.

The use of these devices can ensure different advantages, such as localization at the target site, overcoming the problems related to the BSCB as well as the release of active compounds within a desired range, reducing the side effects of conventional treatments. However, the lack of satisfactory results in SCI treatments is probably due to the fact that they are directed only to single mechanisms, losing sight of the complexity and the multitude of mechanisms involved in SCI. Following this direction, combinatorial treatments represent a new challenge in SCI treatment; thus, the possibility to have simultaneous releases from the same device can be a key point in synergizing the efficacy of multitarget treatments against a multifactorial diseases such as SCI.

## Figures and Tables

**Table 1:** Advantages and disadvantages of the conventional and non-conventional treatments discussed in this review.

		Advantages	Disadvantages
**Conventional**	**Oral**	Non-invasive treatment	Limited access to the SC environment
		Safe and less expensive Possible diffuse treatment of the SC	Metabolism decreases drug in the bloodstream
			Potential side effects
	**Infusion**	Localized immediate pharmacological activity Greater control of drug delivery	Obstruction, leakage, breakage, and dislodgment of catheter
		Rapid reversibility	Possible hemorrhage and infections
		Reduced drug side effects	Limited drug diffusion into the SC
	**Systemic**	Non-invasive treatment	Limited access to the SC environment
		Possible diffuse treatment of the SC	Limited half-life of the drug in the plasma
		Avoid first-pass metabolism	Potential side effects
**Non-Conventional**	**HGs**	Localized and controlled pharmacological activity	Low hydrophobic drug-loading capacity
		High biocompatibility Reduced side effects	Limited control of low-steric-hindrance drug delivery
	**NPs**	Diffuse treatment of the SC	Low hydrophilic drug-loading capacity
		Increased access to the SC environment	Accumulation in organs and macrophages
		Cell-specific targeting	
	**HGs + NPs**	Independent delivery kinetics of different drugs Hydrophobic and hydrophilic drug-loading capacity	Possible elevated uptake of NPs from the microglia
		Localized multi-pharmacological activity	
